# Systemic Hypertension and Predictors of Recanalization After Endovascular Coil Embolization of Intracranial Aneurysms: A Retrospective Cohort Study

**DOI:** 10.3390/jcm15135095

**Published:** 2026-06-30

**Authors:** Jin Byung Park, Su Hwan Lee, Kyuhyuck Lee

**Affiliations:** Department of Neurosurgery, Dongguk University College of Medicine, Dongguk University Ilsan Hospital, 27, Dongguk-ro, Ilsandong-gu, Goyang-si 10326, Gyeonggi-do, Republic of Korea; wlsqud3@naver.com (J.B.P.); kyuhyuck5089@gmail.com (K.L.)

**Keywords:** hypertension, endovascular coil embolization, intracranial aneurysm, recanalization

## Abstract

**Objective**: Systemic hypertension is a well-established risk factor for the development, growth, and rupture of intracranial aneurysms. However, its association with aneurysm recanalization after endovascular coil embolization remains unclear. This study aimed to evaluate the relationship between systemic hypertension and recanalization after coil embolization, and to identify additional risk factors associated with recanalization. **Methods:** We retrospectively analyzed 226 patients with 239 intracranial aneurysms treated by endovascular coil embolization between January 2010 and December 2017. Clinical, angiographic, and procedural variables were assessed. Hypertension was defined according to the Korean Society of Hypertension criteria based on a documented diagnosis or regular antihypertensive treatment. Radiological follow-up was performed primarily with magnetic resonance angiography, with digital subtraction angiography findings reviewed when available. Recanalization was assessed using the Raymond–Roy classification. Hierarchical multivariable logistic regression models were constructed using clinically selected covariates. **Results**: Of 239 aneurysms, 171 (71.5%) were treated in patients with hypertension, and recanalization occurred in 76 (31.8%) during a mean follow-up of 30.9 ± 14.0 months. Hypertension was not independently associated with recanalization (adjusted odds ratio (OR), 0.929; 95% confidence interval (CI), 0.447–1.930; *p* = 0.843). Ruptured presentation, larger aneurysm size, lower packing density, and an initial residual sac were independently associated with recanalization. **Conclusions**: Clinically defined systemic hypertension was not independently associated with recanalization after coil embolization. Ruptured presentation, larger aneurysm size, lower packing density, and an initial residual sac were independently associated with recurrence and may help inform post-treatment surveillance.

## 1. Introduction

Intracranial aneurysms are pathological dilatations of the cerebral arteries caused by degeneration of the internal elastic lamina and media. The prevalence of intracranial aneurysms in the general population is relatively low, ranging from 3 to 5% [1,2]. Endovascular coil embolization has become a widely accepted treatment modality for both ruptured and unruptured aneurysms owing to its minimally invasive nature and favorable clinical outcomes. Yet, despite advances in endovascular techniques, recanalization after coil embolization remains a major limitation, with reported recurrence rates ranging from 20 to 40% [3,4,5]. Recanalization may necessitate retreatment and increase the technical difficulty of subsequent procedures; therefore, identifying factors associated with aneurysm recurrence is clinically important.

Systemic hypertension plays a central role in aneurysm formation, progression, and rupture through vascular remodeling, inflammatory changes, and hemodynamic stress [6,7,8,9]. Chronic hypertension promotes endothelial dysfunction and renin–angiotensin–aldosterone system-mediated vascular remodeling and inflammation [10]. Elevated wall shear stress and altered flow dynamics are associated with aneurysm recurrence after coil embolization [11,12].

Given the close relationship between hypertension and aneurysm pathophysiology, hypertension may also contribute to postembolization recanalization. However, studies that have directly evaluated the association between systemic hypertension and recanalization after coil embolization are limited. Therefore, this study aimed to investigate the relationship between systemic hypertension and recanalization after endovascular coil embolization, and to identify additional risk factors associated with aneurysm recurrence.

## 2. Materials and Methods

### 2.1. Study Design and Patient Population

This retrospective study included patients diagnosed with ruptured or unruptured intracranial aneurysms who underwent endovascular coil embolization at our institution between January 2010 and December 2017. During the study period, 298 patients with 314 aneurysms were screened. Fifteen patients with 17 aneurysms were excluded because the lesions were fusiform or dissecting aneurysms or pseudoaneurysms, or because the patients had undergone retreatment. Among the remaining 283 patients with 297 aneurysms, 57 patients with 58 aneurysms were excluded because they died before follow-up imaging or lacked adequate radiological follow-up within 6 months. The final analysis included 226 patients with 239 aneurysms.

Clinical variables included age, sex, smoking history, alcohol use, hypertension, diabetes mellitus, and hyperlipidemia. Aneurysm and procedural variables included aneurysm size, neck size, depth-to-neck ratio, location, rupture status, stent use, packing density, and immediate postprocedural occlusion status. These variables were abstracted from medical records and angiographic studies.

Hypertension was defined according to the Korean Society of Hypertension criteria [13] as a documented clinical diagnosis, preprocedural use of antihypertensive medication, or a physician-documented diagnosis made during hospitalization or follow-up. Transient blood pressure elevation during the acute presentation of a ruptured aneurysm was not considered diagnostic.

Antihypertensive medication burden was defined by the number of regularly prescribed oral antihypertensive agents, with fixed-dose combination products counted according to their active components. Multidrug therapy was defined as ≥2 agents. In exploratory analyses, blood pressure control was evaluated using available outpatient follow-up records. Uncontrolled hypertension was defined as systolic blood pressure ≥ 140 mmHg and/or diastolic blood pressure ≥ 90 mmHg on repeated measurements, or treatment intensification for inadequate blood pressure control. Patients with insufficient follow-up blood pressure data were classified as having unknown control status.

### 2.2. Radiologic Follow-Up

Immediate postprocedural occlusion status was assessed using digital subtraction angiography (DSA). Routine follow-up imaging was scheduled at 6, 12, 24, and 36 months and was performed primarily using magnetic resonance angiography (MRA). DSA was not part of the routine follow-up protocol; when available, its findings were reviewed alongside MRA findings to support the assessment of occlusion status. Occlusion status was graded according to the Raymond–Roy classification (class I, complete occlusion; class II, residual neck; and class III, residual sac) [14]. Recanalization was defined as worsening of the Raymond–Roy grade on follow-up imaging relative to the immediate postprocedural DSA findings.

### 2.3. Statistical Analysis

Categorical variables were analyzed using the chi-squared test or Fisher’s exact test. Continuous variables were analyzed using Student’s *t*-test. Univariable logistic regression was used to estimate crude odds ratios (ORs) and 95% confidence intervals (CIs) for factors associated with recanalization. All analyses were performed at the aneurysm level, with patient-level variables assigned to each aneurysm. Covariates for the multivariable analyses were selected on clinical grounds rather than by univariable *p*-value screening.

Hypertension, the primary exposure of interest, was retained in all primary multivariable models regardless of its univariable association. Hierarchical logistic regression models were constructed to distinguish baseline clinical and morphological factors from procedural factors. Model 1 included hypertension, age, sex, rupture status, and aneurysm size. Model 2 additionally included stent use, packing density, and initial residual sac. Multicollinearity was assessed using pairwise Pearson correlation coefficients and variance inflation factors (VIFs). Sensitivity analyses were performed by separately excluding packing density and the initial residual sac and by additionally adjusting for neck size.

In exploratory analyses, hypertension status was replaced by either antihypertensive medication burden or follow-up blood pressure control status, while the remaining Model 2 covariates were retained. Non-HTN served as the reference category, and aneurysms with unknown blood pressure control status were excluded from the control-status analysis.

Statistical significance was set at *p* < 0.05. All statistical analyses were performed using SPSS version 25.0 (IBM Corp., Armonk, NY, USA).

## 3. Results

### 3.1. Baseline Characteristics (Table 1)

A total of 226 patients (239 intracranial aneurysms) were included in the final analysis. Multiple aneurysms were identified in 13 patients, whereas eight patients presented with an additional unruptured aneurysm accompanying the ruptured lesion.

**Table 1:** Baseline and aneurysmal characteristics.

Variables		No. (%)	Excluded Due to Inadequate Radiologic Follow Up
Patients (*n*)		226	57
Age (year)		57.9 ± 11.6 ^a^	64 ± 15.8 ^a^
Sex	Female	143 (63.3%)	45 (78.9%)
	Male	83 (36.7%)	12 (21.1%)
Hypertension		162 (71.7%)	33 (57.9%)
Diabetes		45 (19.9%)	10 (17.5%)
Hyperlipidemia		72 (31.9%)	6 (10.5%)
Smoking		87 (38.5%)	17 (29.8%)
Alcohol		82 (36.3%)	10 (17.5%)
Aneurysms (*n*)		239	58
Aneurysm location	Ant. circulation	215 (90.0%)	50 (86.2%)
	Post. circulation	24 (10.0%)	8 (13.8%)
Aneurysm type	Bifurcation	196 (82.0%)	56 (96.6%)
	Side-wall	43 (18.0%)	2 (3.4%)
Aneurysm size (mm)		6.9 ± 3.6 ^a^	7.6 ± 3.3 ^a^
	Small (<10 mm)	195 (81.6%)	43 (74.1%)
	Large (>10 mm)	44 (18.4%)	15 (25.9%)
Neck size (mm)		3.5 ± 1.4 ^a^	4.1 ± 1.7 ^a^
Depth-to-neck ratio		1.5 ± 0.6 ^a^	1.6 ± 0.9 ^a^
Presentation	Ruptured An	124 (51.9%)	47 (81.0%)
	Unruptured An	115 (48.1%)	11 (19.0%)

^a^ Mean ± standard deviation. Ant, anterior; Post, posterior; An, aneurysm.

The mean patient age was 57.9 ± 11.6 (range, 29–82) years, and most patients were female (63.3%). Hypertension was the most common comorbidity (162 patients [71.7%]), followed by hyperlipidemia (31.9%), smoking (38.5%), alcohol consumption (36.3%), and diabetes mellitus (19.9%). Most aneurysms were located at bifurcation sites (82.0%) and measured <10 mm in diameter (81.6%). The mean aneurysm size was 6.9 ± 3.6 mm, with a mean neck diameter and depth-to-neck ratio of 3.5 ± 1.4 mm and 1.5 ± 0.6, respectively.

A total of 57 patients were excluded because adequate radiological follow-up was unavailable. Compared with the final study cohort, the excluded patients were older (64 ± 15.8 years) and more frequently presented with ruptured (81.0%) and large (25.9%) aneurysms.

### 3.2. Aneurysm Distribution (Table 2)

Among the anterior circulation aneurysms (*n* = 215), anterior communicating artery aneurysms were the most common (*n* = 63), followed by posterior communicating artery (*n* = 49), paraclinoid (*n* = 40), and middle cerebral artery aneurysms (*n* = 27). Among posterior circulation aneurysms (*n* = 24), basilar artery aneurysms were the most frequently observed (*n* = 10), followed by superior cerebellar artery aneurysms (*n* = 8). Ruptured aneurysms accounted for 51.9% of all lesions, and were most commonly located in the anterior communicating artery (*n* = 42).

**Table 2:** Classification according to location in coiled aneurysms (*n* = 239).

**Anterior Circulation**	**No. (%)**	**Posterior Circulation**	**No. (%)**
Lesions	215 (90.0%)	Lesions	24 (10.0%)
Acom	63	BA	10
Pcom	49	SCA	8
Paraclinoid	40	PICA	3
MCA	27	VA	2
ACA	16	AICA	1
AchA	10		
ICAB	5		
Ophthalmic	5		
**Ruptured Aneurysm**	**No. (%)**	**Unruptured Aneurysm**	**No. (%)**
Lesions	124 (51.9%)	Lesions	115 (48.1%)
Acom	42	Paraclinoid	32
Pcom	29	Acom	21
MCA	18	Pcom	20
ACA	11	MCA	9
Paraclinoid	8	AchA	7
BA	4	BA	6
AchA	3	ACA	5
SCA	3	SCA	5
VA	2	ICAB	4
ICAB	1	Ophthalmic	4
AICA	1	PICA	2
Ophthalmic	1		
PICA	1		

Acom, anterior communicating artery; Pcom, posterior communicating artery; MCA, middle cerebral artery; ACA, anterior cerebral artery; AchA, anterior choroidal artery; ICAB, internal carotid artery bifurcation; BA, basilar artery; SCA, superior cerebellar artery; VA, vertebral artery; PICA, posterior inferior cerebellar artery; AICA, anterior inferior cerebellar artery.

### 3.3. Radiologic Outcomes (Table 3)

Stent-assisted coiling was performed in 45 (18.8%) aneurysms. The mean packing density was 25.9 ± 7.4%. Favorable occlusion, defined as total occlusion or residual neck, was achieved in 209 aneurysms (87.4%), whereas an initial residual sac was observed in 30 aneurysms (12.6%). During a mean follow-up of 30.9 ± 14.0 months, recanalization occurred in 76 aneurysms (31.8%), with most events detected within the first 12 months after treatment.

**Table 3:** Radiological results in coiled aneurysms (*n* = 239).

Variables			No. (%)
Stent		Yes	45 (18.8%)
		No	194 (81.2%)
Packing density			25.9 ± 7.4% ^a^
Occlusion result	Complete	Total occlusion	171 (71.5%)
		Residual neck	38 (15.9%)
	Incomplete	Residual sac	30 (12.6%)
Recanalization			76 (31.8%)
		At 6 mo	36 (47.4%)
		Within 12 mo	29 (38.2%)
		Within 24 mo	8 (10.5%)
		Within 36 mo	3 (3.9%)
Follow-up period (mo)			30.9 ± 14.0 ^a^

^a^ Mean ± standard deviation. mo, month.

### 3.4. Comparison Between Hypertensive and Nonhypertensive Groups (Table 4)

Recanalization rates were similar in the hypertensive and nonhypertensive groups (31.0% vs. 33.8%; *p* = 0.672). No significant between-group differences were observed in aneurysm size, neck size, rupture status, stent use, packing density, or immediate occlusion status.

**Table 4:** Clinical, aneurysmal, and procedural characteristics of 239 aneurysms according to patient hypertension status.

Variables		HTN Group (*n* = 171)	Non-HTN Group (*n* = 68)	*p*-Value
Sex	Male	69 (40.4%)	16 (23.5%)	0.014
	Female	102 (59.6%)	52 (76.5%)	
Age (years)		58.5 ± 11.6 ^b^	55.8 ± 10.7 ^b^	0.103
Diabetes		33 (19.3%)	15 (22.1%)	0.631
Hyperlipidemia		59 (34.5%)	16 (23.5%)	0.099
Smoking		65 (38.0%)	26 (38.2%)	0.974
Alcohol		67 (39.2%)	19 (27.9%)	0.102
Location	Ant. circulation	154 (90.1%)	61 (89.7%)	0.935
	Post. circulation	17 (9.9%)	7 (10.3%)	
An size (mm)		6.8 ± 3.2 ^b^	7.4 ± 3.1 ^b^	0.212
Neck size (mm)		3.9 ± 1.6 ^b^	3.7 ± 1.3 ^b^	0.294
Depth-to-neck ratio		1.4 ± 0.6 ^b^	1.5 ± 0.6 ^b^	0.394
Aneurysm type	Bifurcation	141 (82.5%)	55 (80.9%)	0.775
	Side wall	30 (17.5%)	13 (19.1%)	
Presentation	Ruptured An	89 (52.0%)	35 (51.5%)	0.936
	Unruptured An	82 (48.0%)	33 (48.5%)	
Stent use		30 (17.5%)	15 (22.1%)	0.420
Packing density (%)		25.4 ± 7.4 ^b^	27.1 ± 7.3 ^b^	0.099
Occlusion result ^a^	Complete	150 (87.7%)	59 (86.8%)	0.841
	Incomplete	21 (12.3%)	9 (13.2%)	
Follow-up period (mo)		30.8 ± 14.3 ^b^	31.2 ± 13.3 ^b^	0.861
Follow-up outcome	Recanalization	53 (31.0%)	23 (33.8%)	0.672
	No recanalization	118 (69.0%)	45 (66.2%)	

Patient-level characteristics were assigned to each aneurysm because the analysis was performed at the aneurysm level. ^a^ Result estimated from Raymond classification. ^b^ Mean ± standard deviation. HTN, hypertension; Ant, anterior; Post, posterior; An, aneurysm; mo, month.

### 3.5. Risk Factors for Recanalization (Table 5)

In univariable logistic regression analysis, larger aneurysm size, larger neck size, bifurcation morphology, ruptured presentation, lower packing density, and an initial residual sac were associated with recanalization. Hypertension was not significantly associated with recanalization in either the clinical/morphological model (Model 1: adjusted OR, 1.023; 95% CI, 0.529–1.979; *p* = 0.946) or the procedural-adjusted model (Model 2: adjusted OR, 0.929; 95% CI, 0.447–1.930; *p* = 0.843). In Model 2, ruptured presentation (adjusted OR, 3.283; 95% CI, 1.591–6.771; *p* = 0.001), larger aneurysm size (adjusted OR per 1 mm increase, 1.210; 95% CI, 1.090–1.344; *p* < 0.001), lower packing density (adjusted OR per 1% increase, 0.922; 95% CI, 0.876–0.969; *p* = 0.002), and an initial residual sac (adjusted OR, 6.757; 95% CI, 2.424–18.841; *p* < 0.001) remained independently associated with recanalization. Pairwise correlations among aneurysm size, rupture status, packing density, and initial occlusion status were modest, with a maximum absolute correlation coefficient of 0.341. VIFs in Model 2 ranged from 1.07 to 1.17. Sensitivity analyses yielded consistent nonsignificant estimates for hypertension when packing density was excluded (adjusted OR, 1.068; 95% CI, 0.528–2.160; *p* = 0.855), when the initial residual sac was excluded (adjusted OR, 0.838; 95% CI, 0.414–1.696; *p =* 0.622), and when neck size was additionally included (adjusted OR, 0.846; 95% CI, 0.402–1.781; *p* = 0.660); the maximum VIF in the neck-size-adjusted model was 2.17.

**Table 5:** Univariable and hierarchical multivariable logistic regression analyses of factors associated with recanalization after coil embolization (*n* = 239 aneurysms).

Variables	Univariable OR (95% CI)	*p*-Value	Model 1 Adjusted OR (95% CI)	*p*-Value	Model 2 Adjusted OR (95% CI)	*p*-Value
Hypertension	0.879 (0.483–1.598)	0.672	1.023 (0.529–1.979)	0.946	0.929 (0.447–1.930)	0.843
Age (per 1-year increase)	1.004 (0.980–1.028)	0.764	0.997 (0.970–1.024)	0.814	0.988 (0.959–1.019)	0.441
Male sex	0.917 (0.518–1.623)	0.765	0.753 (0.394–1.441)	0.392	0.651 (0.312–1.358)	0.253
Diabetes	1.478 (0.769–2.839)	0.241	—	—	—	—
Hyperlipidemia	0.958 (0.534–1.718)	0.885	—	—	—	—
Smoking	1.282 (0.735–2.236)	0.381	—	—	—	—
Alcohol	0.971 (0.550–1.714)	0.920	—	—	—	—
Ant. circulation	1.148 (0.455–2.896)	0.770	—	—	—	—
Aneurysm size (per 1 mm increase)	1.194 (1.094–1.303)	<0.001	1.195 (1.089–1.312)	<0.001	1.210 (1.090–1.344)	<0.001
Neck size (per 1 mm increase)	1.356 (1.132–1.625)	0.001	—	—	—	—
Depth-to-neck ratio (per 1-unit increase)	1.032 (0.645–1.652)	0.895	—	—	—	—
Bifurcation aneurysm	2.794 (1.181–6.610)	0.019	—	—	—	—
Ruptured aneurysm	2.738 (1.542–4.862)	0.001	2.844 (1.537–5.262)	0.001	3.283 (1.591–6.771)	0.001
Stent use	1.389 (0.707–2.731)	0.340	—	—	1.288 (0.538–3.086)	0.570
Packing density (per 1% increase)	0.897 (0.858–0.939)	<0.001	—	—	0.922 (0.876–0.969)	0.002
Initial residual sac	9.671 (3.926–23.826)	<0.001	—	—	6.757 (2.424–18.841)	<0.001

Model 1 included hypertension, age, sex, rupture status, and aneurysm size. Model 2 additionally included stent use, packing density, and initial residual sac. For categorical variables, the reference categories were no hypertension, female sex, no diabetes mellitus, no hyperlipidemia, no smoking, no alcohol use, posterior circulation, side-wall aneurysm, unruptured aneurysm, no stent use, and complete occlusion or residual neck. Odds ratios for continuous variables are expressed per indicated unit increase. OR, odds ratio; CI, confidence interval; Ant, anterior.

### 3.6. Exploratory Analyses of Hypertension Treatment and Control (Table 6)

In exploratory analyses, antihypertensive medication burden and follow-up blood pressure control status were examined as complementary measures of hypertension treatment and control. Neither was significantly associated with recanalization after multivariable adjustment (overall *p* = 0.976 and *p* = 0.931, respectively; Table 6). Compared with the non-HTN group, no significant associations were observed for single-agent therapy, multidrug therapy, controlled hypertension, or uncontrolled hypertension.

**Table 6:** Exploratory analyses of recanalization according to antihypertensive medication burden and blood pressure control status.

Exploratory Hypertension Variable	No. of Aneurysms	Recanalization, *n* (%)	Adjusted OR (95% CI)	*p*-Value
Antihypertensive medication burden				
Non-HTN	68	23 (33.8)	Reference	—
HTN with single-drug therapy	119	34 (28.6)	1.046 (0.412–2.657)	0.925
HTN with ≥2 oral agents	52	19 (36.5)	0.959 (0.413–2.223)	0.922
Follow-up blood pressure control status				
Non-HTN	68	23 (33.8)	Reference	—
Controlled HTN	123	40 (32.5)	1.212 (0.446–3.296)	0.707
Uncontrolled HTN	43	11 (25.6)	1.117 (0.446–2.801)	0.813

Adjusted ORs were estimated using logistic regression models including age, sex, rupture status, an aneurysm size, stent use, packing density, and initial residual sac. The non-HTN group served as the reference in both analyses. Medication burden was defined by the number of regularly prescribed oral antihypertensive agents; fixed-dose combination products were counted according to their active components, and multidrug therapy was defined as ≥2 agents. Five aneurysms with unknown blood pressure control status were excluded from the control-status analysis. The primary HTN variable was not included concurrently with medication burden or control status. HTN, hypertension; OR, odds ratio; CI, confidence interval.

## 4. Discussion

This study examined the association between systemic hypertension and recanalization after endovascular coil embolization of intracranial aneurysms. Although hypertension is a well-established risk factor for aneurysm formation and rupture [6,7,8,9,10], it was not independently associated with recanalization in either hierarchical multivariable model, and the estimates remained consistent across sensitivity analyses. Exploratory analyses of antihypertensive medication burden and follow-up blood pressure control also showed no significant association with recanalization. In contrast, ruptured presentation, larger aneurysm size, lower packing density, and an initial residual sac were independently associated with recurrence. Most recanalization events occurred within the first 12 months after treatment, suggesting that early post-procedural aneurysm stability may be important for the long-term durability of coil embolization.

The role of hypertension in aneurysm formation and rupture may not translate directly to the postembolization setting. Intracranial aneurysm pathobiology involves complex interactions among inflammation, hemodynamics, and vascular remodeling [15]. Systemic hypertension has been associated with altered wall shear stress in the carotid circulation [16] and with the occurrence of cerebral aneurysms [17], while experimental evidence supports a role in aneurysm rupture [18]. After coil embolization, elevated wall shear stress and flow velocity near residual or incompletely occluded portions have been associated with recanalization [11,12], and computational hemodynamic parameters have been shown to predict retreatment [19]. A clinical diagnosis of systemic hypertension may not adequately reflect the local hemodynamic environment within a treated aneurysm. In this cohort, neither the primary analysis nor the exploratory analyses showed a clear association between hypertension and recanalization. However, this finding does not exclude a possible effect of cumulative blood pressure exposure, because medication burden and intermittent outpatient measurements may not fully reflect hypertension duration, adherence, temporal variability, or long-term control.

Aneurysm morphology remained important in determining treatment durability. A larger aneurysm size was independently associated with recanalization, consistent with previous studies reporting higher recurrence rates in large aneurysms [20,21,22]. Greater sac volume and complex geometry may make homogeneous coil distribution and dense packing more difficult, leaving residual spaces that permit persistent inflow and subsequent coil compaction. Neck size was also associated with recanalization in the univariable analysis but was not significant when added to the sensitivity model. Although no meaningful multicollinearity was identified, aneurysm size and neck size may capture overlapping morphological information. Wide-neck aneurysms nevertheless remain technically challenging because stable coil placement may be difficult and the risk of coil protrusion may limit packing at the aneurysm neck [23,24].

Lower packing density and an initial residual sac were independently associated with recanalization. A loosely packed coil mass may permit persistent intra-aneurysmal flow and progressive coil compaction, whereas an initial residual sac may maintain a channel for continued inflow and hemodynamic stress. These findings are consistent with previous angiographic follow-up studies linking residual aneurysm filling and incomplete occlusion with subsequent recurrence [20,25]. A prospective multicenter cohort study by Pierot et al. identified ruptured status, larger aneurysm size, and wide neck as factors associated with recanalization after coiling [26], while long-term angiographic follow-up has also documented recurrent filling in treated aneurysms [27]. Aneurysm size, rupture status, packing density, and initial occlusion are clinically interrelated, because aneurysm morphology and presentation may influence procedural strategy and the immediate angiographic result. Although the hierarchical models, low VIFs, and sensitivity analyses did not indicate problematic statistical collinearity or materially alter the hypertension estimates, the causal relationships among these factors cannot be determined from this retrospective study. Packing density and initial residual sac should therefore be interpreted primarily as markers of treatment durability rather than as proven causal determinants of recurrence.

Ruptured presentation also remained independently associated with recanalization. In this study, recanalization occurred in 52 of 124 ruptured aneurysms (41.9%). Similar findings have been reported in previous angiographic follow-up studies [21,28]. Several mechanisms may contribute to this association. Thrombus incorporated into the coil mass during the acute phase may subsequently undergo resorption, creating space for coil compaction or recurrent filling. A more conservative packing strategy may also be selected in ruptured aneurysms because of concerns regarding intraprocedural rupture or early rebleeding. In addition, ruptured aneurysms may have greater wall fragility and inflammatory changes that reduce the durability of occlusion [29].

From a clinical perspective, postembolization surveillance should not be based on hypertension status alone. Aneurysms with larger size, ruptured presentation, lower packing density, or an initial residual sac may warrant closer radiological follow-up, particularly during the first year after treatment, when most recanalization events were detected. Incorporating these aneurysm and procedural characteristics into follow-up planning may facilitate earlier recognition of recurrence and timely consideration of retreatment.

Future prospective multicenter studies should incorporate standardized longitudinal blood pressure assessment, including home or ambulatory monitoring, detailed information on antihypertensive treatment and adherence, and uniform imaging follow-up. Such studies may clarify whether cumulative blood pressure exposure influences recanalization and help validate risk-adapted surveillance strategies based on aneurysm and procedural characteristics.

The present study has some limitations. First, the retrospective, single-center design and the exclusion of approximately one-fifth of the eligible cohort because of death or inadequate imaging follow-up may have introduced selection bias. The excluded patients were older and more frequently had ruptured or large aneurysms, suggesting that loss to follow-up was related to clinical severity rather than occurring at random. Consequently, the observed recanalization rate may not fully represent the entire treated population. Second, hypertension treatment and control were assessed retrospectively. Medication count may not adequately reflect hypertension severity, duration, adherence, or treatment changes. Because blood pressure control was assessed during follow-up, its temporal relationship with recanalization could not be clearly established. Home and ambulatory blood pressure monitoring were not systematically available. Third, procedures were performed by multiple operators, and variation in coil selection, packing strategy, and procedural decision-making may have influenced the outcomes. Finally, the analyses were performed at the aneurysm level, and potential within-patient correlation among multiple aneurysms was not explicitly modeled. Despite these limitations, the study provides long-term radiological follow-up and an integrated assessment of clinical, morphological, procedural, and hypertension-related factors.

## 5. Conclusions

Clinically defined systemic hypertension was not independently associated with recanalization after endovascular coil embolization, and this lack of association was also observed in exploratory analyses of medication burden and follow-up blood pressure control. Recanalization was instead associated with ruptured presentation, larger aneurysm size, lower packing density, and an initial residual sac. Post-treatment surveillance may therefore be better guided by aneurysm and procedural characteristics than by hypertension status alone.

## Data Availability

Data used in this study can be provided upon reasonable request.

## Abbreviations

The following abbreviations are used in this manuscript:

CI	Confidence interval
DSA	Digital subtraction angiography
HTN	Hypertension
MRA	Magnetic resonance angiography
OR	Odds ratio
VIF	Variance inflation factor
